# Investigating the Potential of Acacia nilotica-Enriched Glass Ionomer Cement: An Analysis of Antimicrobial Activity and Compressive Strength

**DOI:** 10.7759/cureus.54821

**Published:** 2024-02-24

**Authors:** Jessy Paulraj, Jeyashree T, Yuvashree C S, Rajeshkumar Shanmugam, Subhabrata Maiti

**Affiliations:** 1 Department of Pedodontics and Preventive Dentistry, Saveetha Dental College and Hospitals, Saveetha Institute of Medical and Technical Sciences, Saveetha University, Chennai, IND; 2 Nanobiomedicine Lab, Saveetha Medical College and Hospital, Saveetha Institute of Medical and Technical Sciences, Chennai, IND; 3 Department of Prosthodontics, Saveetha Dental College and Hospitals, Saveetha Institute of Medical and Technical Sciences, Saveetha University, Chennai, IND

**Keywords:** restorative dentistry, compressive strength, antimicrobial activity, modified gic, acacia nilotica

## Abstract

Background

According to existing literature, introducing natural antibacterial agents into glass ionomer cement (GIC) has been associated with potential negative impacts on their strength properties. Hence, this study aims to explore the antibacterial effectiveness of glass ionomer cement enriched with *Acacia nilotica* and subsequently assess its compressive strength characteristics.

Aim

The objective of the study is to assess the antimicrobial effectiveness and compressive strength of glass ionomer cement modified with *Acacia nilotica*.

Materials and methods

The plant extract was incorporated into the conventional glass ionomer cement in three different proportions (powder ^GIC^: extract: liquid ^GIC^), divided into group I, group II, and group III with ratios of 2:1:1, 3:1:2, and 3:2:1 respectively. Additionally, a control group denoted as group IV was included without any modifications. Subsequently, the specimens were prepared, and their chemical structure was analyzed using Fourier transform infrared spectroscopy (FTIR), followed by testing for antimicrobial activity using the minimum inhibitory concentration (MIC) assay against *Streptococcus mutans *and *Lactobacillus*. The assessment of compressive strength was conducted following ISO 9917-1:2007 standards, and the recorded values represent the maximum force the specimen could withstand before fracturing.

Results

The antimicrobial effectiveness against *Streptococcus mutans* and *Lactobacillus *exhibited a notable increase in all modified specimens compared to the control group, with a significance level of p<0.05. Additionally, significant improvements in compressive strength were observed in group III (183.49±2.99) when compared to the remaining groups. The higher concentrations of the plant extract resulted in superior outcomes.

Conclusion

Therefore, the incorporation of *Acacia nilotica* into GIC shows promising potential as a restorative material. These investigations can provide valuable insights into the material's performance and durability, contributing to its potential application in dental restorations. Future research is needed to thoroughly investigate the bonding chemistry between* Acacia nilotica* and GIC, as well as to assess the extent of microleakage.

## Introduction

Glass ionomer cement (GIC) is categorized as acid-base cement, commonly referred to as glass ionomer in dentistry [1]. In recent times, glass ionomer cements (GICs) have been widely employed for the final cementation of dental crowns and fillings [2]. The key attributes that contribute to their popularity include adhesion to the tooth, biocompatibility, and the controlled release of fluoride ions. Additionally, studies have revealed the potential for GICs to be recharged with fluoride ions [3,4]. Despite their favorable qualities, bacteria have been found to remain viable under GIC for up to two years [5]. Moreover, within the initial hours following placement into the cavity, GICs produce a lower level of fluoride, potentially being inadequate for achieving the desired bactericidal effects [6,7]. Recognizing the therapeutic advantages of incorporating antimicrobial agents into GIC prompted the modification of GIC. Unfortunately, the addition of antibacterial agents to restorative materials often leads to adverse effects on their physical and mechanical properties over time [8,9]. Poorly managed doses or releases may result in short-term effectiveness and potential hazards to nearby tissues. This may explain the reluctance to incorporate GICs in production when combined with chlorhexidine and triclosan [10,11].

Plants were utilized for preventive and therapeutic purposes for centuries, predating the emergence of iatrochemistry in the 16th century [12]. Phytomedicine, a form of herbal medicine, involves the utilization of different plant components or extracts for medicinal or health-enhancing purposes. The use of herbal extracts offers the advantage of yielding positive outcomes. *Acacia nilotica* trees, commonly referred to as "babool" and also known by other names such as kikar powder, babool chaal powder, babul chhaal powder, *Acacia arabica*, baval, keekar, kikar, babul, babool, babla, and babur. These trees have been traditionally used in Unani and various Indian systems of medicine for the prevention and treatment of diverse illnesses. *Acacia nilotica* belongs to the Fabaceae family within the Plantae kingdom [13]. As the second-largest genus in the Fabaceae family, it comprises over 1350 species and is globally distributed around regions like Asia, Africa, and America. *Acacia nilotica* is rich in complex phytoconstituents, including alkaloids, flavonoids, and terpenes [14].

Different components of the acacia tree, such as leaves, bark, seeds, etc., have traditionally been employed for both nutrition, as well as medical purposes to prevent and treat various ailments. Acacia is particularly abundant in antioxidant phenolics, specifically condensed tannins and phlobatannins. The distinct phytoconstituents present in *Acacia nilotica*, including antibacterial and antiplaque properties, contribute to its medicinal effects. According to traditional descriptions, *Acacia nilotica* holds significant medicinal value and is known for its ability to prevent, reduce, and treat various infectious diseases and adverse conditions. While certain products like mouthwash or toothpaste have shown effectiveness against cariogenic salivary flora, there is a lack of comprehensive information in existing literature concerning the integration of natural antibacterial agents into glass ionomer cements (GICs). Hence, the objective of this study was to enhance the antimicrobial features of glass ionomer cement by incorporating a blend of *Acacia nilotica* extracts. Subsequently, these modified GICs were compared to a control GIC for both microbial and compressive strength properties.

## Materials and methods

Extract preparation 

**Figure 1:**
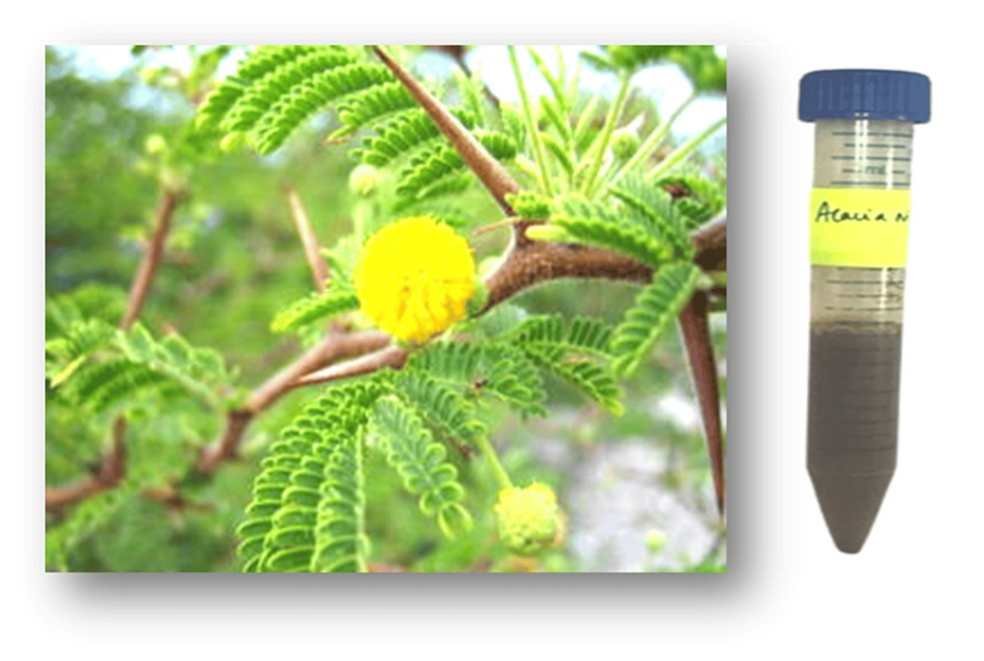
Acacia nilotica leaves and extract

Leaves were air-dried for five days. Glassware was thoroughly cleaned and dried at 70 degrees Celsius. One gram of dried leaves was added to 100 mL distilled water in a beaker, and boiled at 60-70 degrees Celsius for 15 minutes. The filtered solution (80 mL) was concentrated to 5 mL at 60-70 degrees Celsius (Figure 1).

Inoculum preparation

Bacterial strains (*Streptococcus mutans* and *Lactobacillus acidophilus*) were acquired from the Department of Microbiology, cultured on Mueller Hinton agar using a sterile loop, subcultured, and individually inoculated into tubes with 5 mL of sterile Mueller Hinton broth. After 24 hours of incubation at 37 degrees Celsius, the suspension was adjusted to a 0.5 McFarland scale.

Sample preparation

The plant extract was added to the conventional GIC mixture, and the resulting cement was placed in cylindrical molds (6 mm diameter, 2 mm thickness). After the setting of cement, the specimens were de-molded, and their precise dimensions were measured using calipers. Standard strains of *Streptococcus mutans *and *Lactobacillus *were utilized to assess the antibacterial effects. For each group, twelve specimens were distributed, with six assigned for *Streptococcus mutans *testing and six for *Lactobacillus *evaluation. To assess compressive strength, cylindrical molds (4.0 mm diameter, 6.0 mm height) were used per ISO 9917-1:2007 guidelines. Six specimens per group were crafted, ensuring a smooth surface. After an hour, specimens were removed and soaked in deionized water for 24 hours, and defective ones were discarded.

Fourier transform infrared (FTIR) spectroscopy analysis

FTIR spectroscopy was employed to analyze and characterize the molecular components and structures of the *Acacia nilotica* modified glass ionomer cement. The chemical structure of the specimens was assessed using a spectrometer (Nicolet iS10; Thermo Fisher Scientific, Waltham, Massachusetts), with the fine powder compressed into a uniform disc for examination, and data were collected within the range of 500-4000 cm−1.

Determination of antimicrobial activity

To evaluate the antimicrobial efficacy of both altered and unaltered GIC, standard strains of *Streptococcus mutans *and *Lactobacillus *were employed. The wells were filled with 200 µL of sterilized MHA broth, after which 50 µL of bacterial suspensions (*Streptococcus mutans *and *Lactobacillus acidophilus*) with a concentration of 5×105 CFU/ml were added. The samples from groups I, II, III, and IV were introduced into the incubating wells and labeled accordingly. Incubation was carried out under appropriate conditions for different durations (one hour to four hours). The percentage of dead cells was determined using an enzyme-linked immunosorbent assay (ELISA) reader at a wavelength of 540 nm.

Determination of compressive load

**Figure 2:**
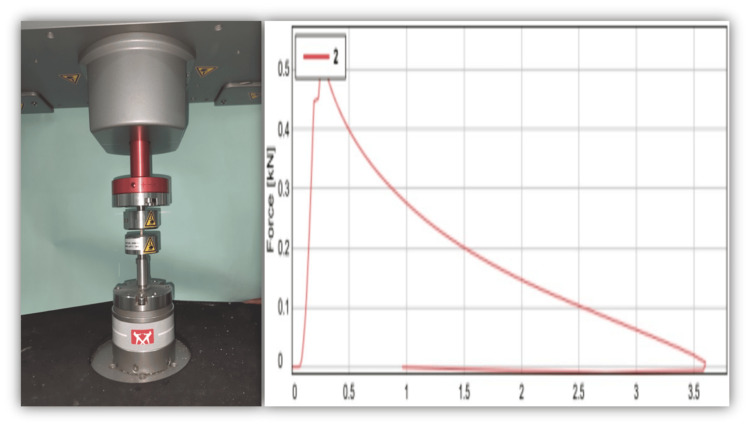
Compressive strength testing The X-axis represents displacement (mm) and the Y-axis represents force (N)

After preparation, specimens were vertically placed in a Universal Testing Machine (ElectroPuls® E3000; Instron, Norwood, Massachusetts). A compressive load was exerted along the long axis at a crosshead speed of 0.5 mm/min until fracture. The maximum force observed at the point of fracture was noted, and the compressive strength values were subsequently calculated (Figure 2).

Statistical analysis

Collected data was entered into an Excel spreadsheet (Microsoft, Redmond, Washington), and statistical analysis utilized SPSS version 24.0 (IBM Inc., Armonk, New York). Descriptive analysis and repeated measure ANOVA determined mean minimum inhibitory concentration (MIC) values. Compressive strength comparisons employed one-way ANOVA, with Tukey's post hoc test for pairwise comparisons at p≤0.05 and 95% confidence intervals.

## Results

FTIR spectra

**Figure 3:**
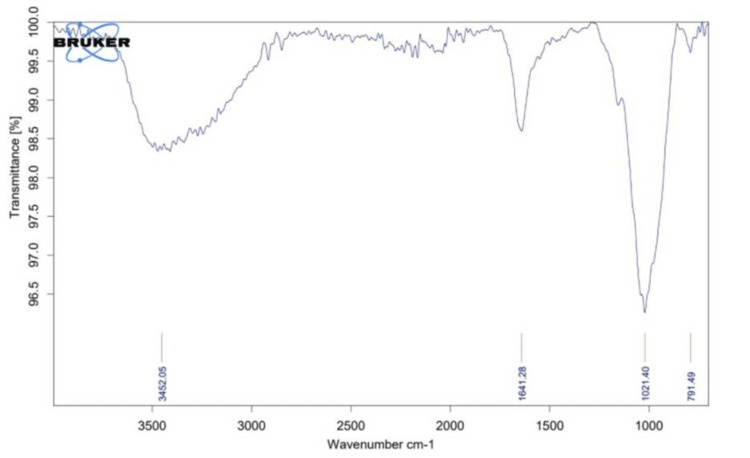
FTIR spectra of Acacia nilotica modified GIC FTIR - Fourier transform infrared; GIC - glass ionomer cement

**Figure 4:**
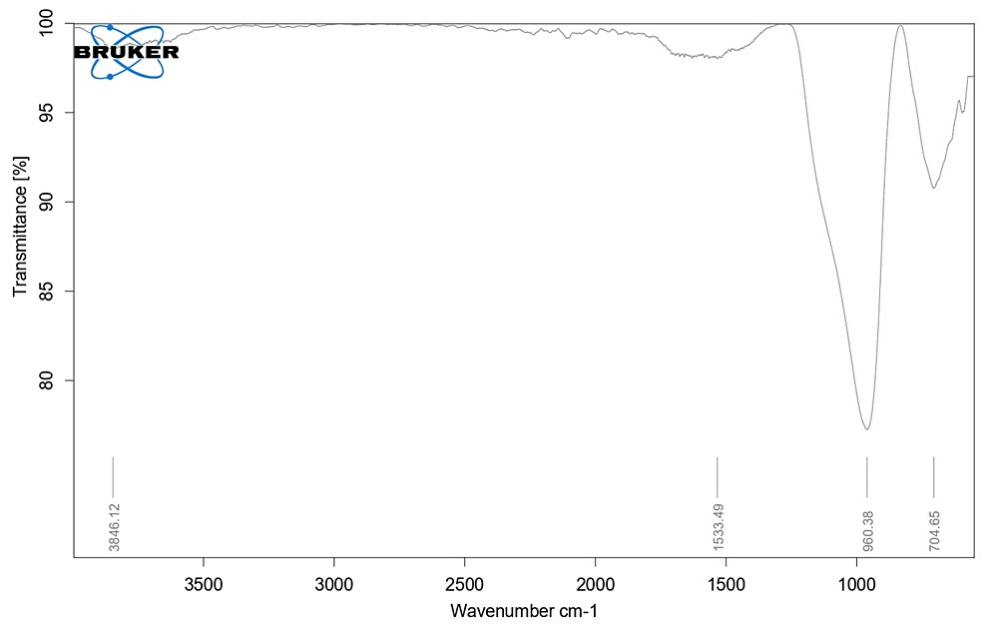
FTIR spectra of conventional GIC FTIR - Fourier transform infrared; GIC - glass ionomer cement

The FTIR spectrum indicates a slight shift in peaks in the modified sample (Figure 3) when compared with the unmodified sample (Figure 4).

The increased absorbance at 1021 cm−1 may be attributed to C-O stretching, while the absorption peak at 1641 cm−1 could be associated with C=O vibration, potentially linked to the presence of aldehyde groups. Additionally, the absorption peak at 3452 cm−1 may correspond to the OH stretching of the acid groups. These changes in peak sharpness and shift within the chemical structure before and after the modification of GIC could be a result of chemical interactions between the *Acacia nilotica* extract and Glass ionomer cement.

Antimicrobial activity

**Figure 5:**
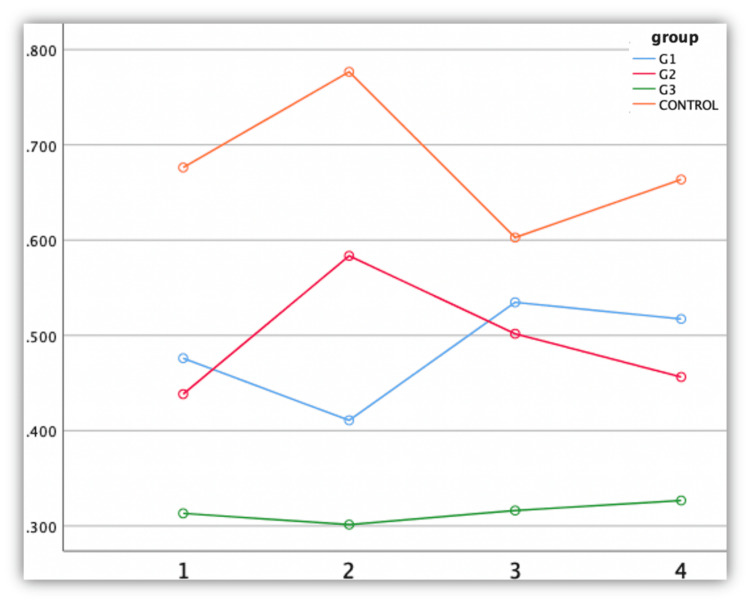
Antimicrobial efficacy against Streptococcus mutans The X-axis represents time interval (hours) and the Y-axis represents optical density

In this study, repeated measure ANOVA assessed the antibacterial effects of modified and unmodified GIC against *Streptococcus mutans*. *Acacia nilotica* modification showed better performance, significantly outperforming group IV (control) (Figure 5).

The antimicrobial activity of group III (3:2:1) *Acacia nilotica* modification showed the best outcome (mean value: 0.301). The Tukey honestly significant difference (HSD) multiple comparison test revealed a significant difference between group IV and the other three groups (p<0.05) (Table 1).

**Table 1:** Pairwise comparison of antimicrobial efficacy on Streptococcus mutans between four groups *P-value was significant at 0.05, p-value was derived from multiple comparison Tukey honestly significant difference test.

Pairwise comparison	Mean difference	95% CI		p-value
		Lower	Upper	
Group I vs. group II	0.010	0.009	0.011	0.001*
Group I vs. group III	0.170	0.169	0.171	0.001*
Group I vs. group IV	0.195	0.194	0.195	0.001*
Group II vs. group III	0.180	0.179	0.181	0.001*
Group II vs. group IV	0.184	0.184	0.185	0.001*
Group III vs. group IV	0.365	0.364	0.366	0.001*

**Figure 6:**
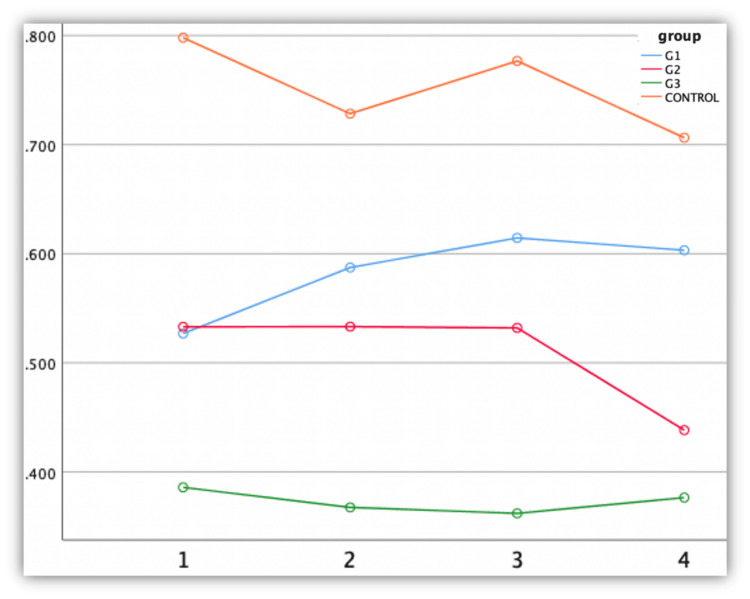
Antimicrobial efficacy against Lactobacillus The X-axis represents time interval (hours) and the Y-axis represents optical density

Modified *Acacia nilotica* groups showed significantly better antimicrobial activity against lactobacillus compared to the control group (group IV) (Figure 6).

The higher concentrations of the plant extract resulted in superior outcomes against Lactobacillus, with the 3:2:1 concentration yielding the lowest mean value of 0.361. The Pairwise comparison confirms the significant difference between group IV and other groups (p<0.05), highlighting the enhanced antibacterial potential of *Acacia nilotica*-modified groups over conventional controls (Table 2).

**Table 2:** Pairwise comparison of antimicrobial efficacy on Lactobacillus between four groups *P-value was significant at 0.05, p-value was derived from multiple comparison Tukey honestly significant difference test.

Pairwise comparison	Mean difference	95% CI		p-value
		Lower	Upper	
Group I vs. group II	0.073	0. 073	0.074	0.001*
Group I vs. group III	0.210	0.209	0.211	0.001*
Group I vs. group IV	0.169	0.168	0.170	0.001*
Group II vs. group III	0.136	0.135	0.137	0.001*
Group II vs. group IV	0.243	0.242	0.244	0.001*
Group III vs. group IV	0.379	0.378	0.381	0.001*

Compressive strength

Specimens underwent compression loading, and linear graph values were recorded. A one-way analysis of variance (ANOVA) revealed a statistically significant difference in compressive strength among the groups, with an F value of 42.67 and a p-value of 0.001 (p<0.05) (Table 3).

**Table 3:** Comparison between groups for evaluation of compressive strength *Significant at 0.05, p-value was derived by one-way ANOVA

Group	n	Mean ± SD	95 % CI		F value	p-value
			Lower	Upper		
Group I	12	174.47±1.81	173.31	175.61	42.67	0.001*
Group II	12	176.91±2.20	175.51	178.31		
Group III	12	183.49±2.99	181.59	185.39		
Group IV	12	173.29±2.51	171.69	174.88		

Tukey's post hoc test for pairwise comparison indicated a significant difference between group III when compared with other groups (p<0.05), affirming that group III, which has higher extract concentrations, exhibited greater compressive strength compared to the other group (Table 4).

**Table 4:** Pairwise comparison for evaluation of compressive strength *significant difference at p=0.05, p-value was derived from Tukey Post hoc test

Pairwise comparison	Mean difference	95% CI		p-value
		Lower	Upper	
Group I vs. group II	2.45	0.184	5.084	0.077
Group I vs. group III	9.02	6.39	11.65	0.001*
Group I vs. group IV	1.17	1.45	3.80	0.636
Group II vs. group III	6.57	3.94	9.20	0.001*
Group II vs. group IV	3.62	0.99	6.25	0.013
Group III vs. group IV	10.20	7.56	12.83	0.001*

## Discussion

Dental caries is a multifactorial disease that affects both primary and permanent teeth, causing damage to tooth crowns and exposed root surfaces [15]. Despite being preventable, dental caries imposes significant economic and quality-of-life burdens [16]. Glass-ionomer cement (GIC) has been extensively used in dentistry for over four decades, primarily valued for its long-term antimicrobial release. However, challenges persist in their use as restorative materials, including susceptibility to secondary caries and low mechanical properties. Recent studies indicate that the fluoride-releasing capability of conventional GICs may be insufficient for effective antibacterial conservation in many cases [17]. Consequently, various efforts have been made to enhance the antibacterial properties of GICs to prevent secondary caries. Incorporating GICs into biomaterials with inherent antibacterial activities has been explored for this purpose. While studies have demonstrated the therapeutic benefits of incorporating antibacterial agents into restorative materials, this often comes at the cost of compromised physical and mechanical properties [18]. This limitation underscores the need for innovative strategies, such as exploring the antimicrobial properties of readily available medicinal plants, to combat dental caries without compromising material integrity. In this study, *Acacia Nilotica* extract was incorporated into the liquid components of glass ionomer cement (GIC) at three distinct concentrations. The resultant modifications were evaluated and contrasted with a conventional GIC regarding their antimicrobial activity and compressive strength characteristics. The antimicrobial effectiveness was assessed using the minimum inhibitory concentration (MIC) assay, which specifically targeted *Streptococcus mutans *and *Lactobacillus *due to their pivotal involvement in dental caries [19].

In the present study, MIC assay results against *Streptococcus mutans *and *Lactobacillus *indicated a substantial inhibitory effect on bacterial growth in the modified group compared to the others. As the concentration of the extract heightened, this effect became more prominent. These findings align with the observations of Becci et al., who emphasized the concentration-dependent nature of antimicrobial activity [20]. Also, a study done by Nagumanthri et al. investigated the antimicrobial activity of *Acacia nilotica* and identified it as having the most potent antimicrobial extract [21]. Mahesh and Satish explored the significant antibacterial activity of methanol leaf and bark extracts of *Acacia nilotica* against *Bacillus subtilis*, *Escherichia coli*, and *Staphylococcus aureus* [22]. Hassan et al. tested the antimicrobial activity of the ethanolic extract of *Acacia arabica* in vitro against seven bacterial species, demonstrating the effectiveness of ethanolic extracts on bacterial strains [23]. Jodi et al. studied the antimicrobial activity of *Acacia nilotica* against medicinally important bacterial strains [24]. An in vitro study assessed the effectiveness of 5%, 10%, and 50% extracts of dried chewing sticks of *Acacia nilotica* on *Streptococcus mutans*, revealing the impact of various concentrations of aqueous *Acacia nilotica* extracts on *Streptococcus mutans *[25]. In a double-blind, randomized control trial, Gupta et al. evaluated the clinical effects of three mouth rinses against salivary *Streptococcus* *mutans*, with the antibacterial action of *Acacia nilotica* comparable to that of chlorhexidine [26]. Thus, the antibacterial property of *Acacia nilotica* is attributed to tannins, phenolic compounds, essential oils, and flavonoids. There is growing evidence supporting the richness of bioactive secondary compounds in acacia plants, making them promising for drug discovery. Secondary compounds in acacia, such as triterpenoids, saponins, glucosides, polysaccharides, gum, tannins, flavonoids, polyphenols, tryptamine, and organic acids, play important roles. The flavonoids present in the flower, fruit, and leaves are key constituents responsible for the antimicrobial property, exhibiting roles in inhibiting microbial growth, cytoplasmic membrane function, attachment and biofilm formation inhibition, and membrane permeability alteration [27]. Previous literature has focused on the efficacy of individual components of *Acacia nilotica*. However, this study represents the first evaluation of *Acacia nilotica* as a restorative material by incorporating it with glass ionomer cement. The current investigation demonstrates that *Acacia nilotica*-enriched GIC exhibits high antimicrobial potency against *Streptococcus mutans *and *Lactobacillus*.

The incorporation of novel compositions in the development of glass ionomer cement (GIC) holds great promise for enhancing the clinical performance of tooth restorations, particularly in areas exposed to high mastication forces, such as the occlusal surfaces of posterior teeth. Compressive strength is a widely used parameter to characterize dental cement, as stress and strain play a major role during mastication [28]. Therefore, assessing the compressive strength becomes crucial when modifying GIC. In this study, the compressive strength characteristics of *Acacia nilotica*-modified GIC were significantly higher than conventional GIC, aligning with the findings of Khan et al., who stated that the addition of gum arabic from *Acacia seyal* to GIC powder (luting cement) resulted in increased compressive strength characteristics [29]. The increased compressive strength can be credited to *Acacia nilotica*, which harbors water-soluble polysaccharides capable of creating hydrogen bonds with the polyacrylic acid constituent of the cement. Furthermore, the existence of calcium ions within *Acacia nilotica* can engage with glass particles within the cement, leading to a more robust bond and enhancing both the cement's strength and its overall mechanical characteristics. [30]. Another study by Singer et al. also supported our results, concluding that the higher concentrations of the plant extract led to enhancements in compressive strength [28].

Based on the aforementioned results, it can be concluded that an increased concentration of *Acacia nilotica* extract has the potential to improve both antimicrobial efficacy and compressive strength. This has clinical significance as it may inhibit the growth of *Streptococcus mutans* and *Lactobacillus*, thereby impeding the progression of caries and reducing the risk of restoration failure. This approach could be beneficial in clinical applications, as it has the potential to reduce the risk of secondary caries with the ability to withstand mastication forces. Future research endeavors should focus on evaluating its cytotoxicity, shear bond strength, and molecular composition. The study's limitation is the lack of sufficient sample size or diversity in experimental conditions. Furthermore, extensive research is required to evaluate the cytotoxicity, bond strength, wear resistance, and molecular chemistry for more robust and comprehensive results. 

## Conclusions

*Acacia nilotica*-modified GIC exhibits increased antimicrobial efficacy and compressive strength at elevated concentrations, suggesting its potential as a promising restorative material with broader benefits yet to be fully understood. Further investigations are needed to evaluate its cytotoxicity, shear bond strength, and molecular chemistry.
